# Two subtypes of cutaneous melanoma with distinct mutational signatures and clinico-genomic characteristics

**DOI:** 10.3389/fgene.2022.987205

**Published:** 2022-09-29

**Authors:** Yoon-Seob Kim, Minho Lee, Yeun-Jun Chung

**Affiliations:** ^1^ Department of Microbiology, College of Medicine, The Catholic University of Korea, Seoul, South Korea; ^2^ Precision Medicine Research Center, College of Medicine, The Catholic University of Korea, Seoul, South Korea; ^3^ Integrated Research Center for Genome Polymorphism (IRCGP), College of Medicine, The Catholic University of Korea, Seoul, South Korea; ^4^ Department of Life Science, Dongguk University-Seoul, Goyang-si, Gyeonggi-do, South Korea; ^5^ Department of Biomedicine and Health Sciences, College of Medicine, The Catholic University of Korea, Seoul, South Korea

**Keywords:** cutaneous melanoma, mutational signature, next generation seqencing, ultraviolet, melanoma

## Abstract

**Background:** To decipher mutational signatures and their associations with biological implications in cutaneous melanomas (CMs), including those with a low ultraviolet (UV) signature.

**Materials and Methods:** We applied non-negative matrix factorization (NMF) and unsupervised clustering to the 96-class mutational context of The *Cancer* Genome Atlas (TCGA) cohort (*N* = 466) as well as other publicly available datasets (*N* = 527). To explore the feasibility of mutational signature-based classification using panel sequencing data, independent panel sequencing data were analyzed.

**Results:** NMF decomposition of the TCGA cohort and other publicly available datasets consistently found two mutational signatures: UV (SBS7a/7b dominant) and non-UV (SBS1/5 dominant) signatures. Based on mutational signatures, TCGA CMs were classified into two clusters: UV-high and UV-low. CMs belonging to the UV-low cluster showed significantly worse overall survival and landmark survival at 1-year than those in the UV-high cluster; low or high UV signature remained the most significant prognostic factor in multivariate analysis. The UV-low cluster showed distinct genomic and functional characteristic patterns: low mutation counts, increased proportion of triple wild-type and *KIT* mutations, high burden of copy number alteration, expression of genes related to keratinocyte differentiation, and low activation of tumor immunity. We verified that UV-high and UV-low clusters can be distinguished by panel sequencing.

**Conclusion:** Our study revealed two mutational signatures of CMs that divide CMs into two clusters with distinct clinico-genomic characteristics. Our results will be helpful for the clinical application of mutational signature-based classification of CMs.

## Introduction

Cancers can arise as a result of somatic mutations caused by mutational processes of both exogenous and endogenous origins. These mutations are imprinted in cancer genomes and characterized by mutational signatures (Stratton et al., 2009; Alexandrov et al., 2020). To date, 60 single base substitution-based mutational signatures have been defined in the Catalogue Of Somatic Mutations In *Cancer* (COSMIC) database (Tate et al., 2018). However, some signatures that are rare or geographically restricted, those conferring limited mutation burdens, and those representing therapeutic mutagenic exposure still require clarification (Alexandrov et al., 2020). Recent studies have revealed that mutational signature analysis can provide not only footprints for exposure to environmental factors and infidelity of DNA replication and repair pathways, but also therapeutic implications of specific drug responses; thus, mutational signature analysis can be a promising tool for molecular cancer diagnosis, treatment choice, and prognostic prediction (Kim et al., 2016; Wang et al., 2018; Gulhan et al., 2019; Staaf et al., 2019; Färkkilä et al., 2020; Abbasi and Alexandrov, 2021; Chong et al., 2021).

Cutaneous melanoma (CM) has the highest mutational burden among common cancers (Cancer Genome Atlas Network, 2015). CM mutational signatures are mainly caused by ultraviolet (UV) radiation, which induces DNA damage, including predominant C-to-T nucleotide transitions at dipyrimidine sites, and microenvironmental alterations (Craig et al., 2018). Predominant UV signatures and a minor proportion of age-related signatures have been reported in CM (Alexandrov et al., 2020). Previous studies have shown that clinico-genomic characteristics, such as age at diagnosis, mutational burden, and frequency of driver mutations, are different between chronically sun-damaged skin and unaffected areas (Craig et al., 2018; Shain et al., 2018; Ghiasvand et al., 2019). Acral and mucosal melanomas are known to harbor low UV signatures and distinct clinico-genomic features (Hayward et al., 2017; Rabbie et al., 2019; Newell et al., 2020). Recent genomic studies have revealed that CMs with a high UV signature typically display better prognosis (Trucco et al., 2019; Vicente et al., 2022) and immunotherapeutic outcomes (Pham et al., 2020; Dousset et al., 2021). Recently, a multi-omics study revealed that CMs with a low UV signature harbored distinct epigenetic profiles in regulatory regions and immunological pathways resembling acral melanoma (Vicente et al., 2022). However, the mutational signatures and clinico-genomic characteristics of CMs with low UV signatures have not been fully characterized yet.

The purpose of our study was to decipher mutational signatures in CM and investigate their clinical associations. Using The *Cancer* Genome Atlas (TCGA) and other publicly available datasets of CM, we identified two clusters of CM with distinct clinico-genomic characteristics. We also explored whether the classification of CM based on the mutational signature is feasible using panel sequencing.

## Materials and methods

### Study dataset

We used TCGA whole-exome sequencing (WES) and other publicly available datasets (Supplementary Table S1). For TCGA dataset (*N* = 466) (2015), clinical information and curated somatic mutation data (public MAF file generated from MuTect2) were downloaded (https://portal.gdc.cancer.gov/). For the International *Cancer* Genome Consortium (ICGC) dataset (*N* = 235), the clinical information and curated somatic mutations of two CM cohorts (Hayward et al., 2017; Campbell, 2020) were downloaded (https://dcc.icgc.org/). Raw sequence reads from seven independent studies (Krauthammer et al., 2012; Snyder et al., 2014; Hugo et al., 2016; Liang et al., 2017; Riaz et al., 2017; Roh et al., 2017; Shain et al., 2018) were downloaded and processed for the Sequence Read Archive (SRA) dataset (*N* = 292). Patients with fewer than five single nucleotide variants were excluded from the analysis. Details are available in the Supplementary Methods.

### De novo mutational signature extraction and unsupervised clustering

We used the ‘sigprofilerextractor’ function in the ‘SigProfilerExtractor’ R package to define the most appropriate number of mutational signatures active in CM (Islam et al., 2022). Based on the metrics of average sample cosine distance and average silhouette width plotted across a range of ranks (2 to 8) calculated by the tool, the proper rank of signature decomposition was considered to be two (Supplementary Figure S1). *De novo* mutational signatures were extracted from a 96-class mutational context using the ‘nmf’ function in the non-negative matrix factorization (NMF) R package (Gaujoux and Seoighe, 2010), using the following parameters: “rank = 2, nrun = 1,000, method = ‘brunet’”. The NMF decomposition of the 96-mutational class was performed to determine the two mutational signatures and the weight of each signature. Unsupervised k-means clustering of the coefficient matrix from NMF was performed.

### Signature refitting analysis

Mutational signatures were analyzed by linear decomposition using the DeconstrucSigs package (Rosenthal et al., 2016). The relative contributions of mutational signatures were calculated by refitting the linear combinations of COSMIC v3 signatures identified in the WES studies of CM (Alexandrov et al., 2020) (details are available in Supplementary Methods) or NMF-extracted signatures (SigA and SigB). Cosine similarity was calculated as described previously (Gulhan et al., 2019) and used to evaluate the similarity between mutational signatures as well as the accuracy of signature decomposition.

### Survival analysis

The survival data of the patients in the TCGA and ICGC datasets were analyzed using various tests. A Kaplan–Meier survival analysis and two-tailed log-rank test were performed using the ‘survminer’ and ‘survival’ packages. For multivariate survival analysis of variables affecting overall survival, Cox proportional hazards model and regression analyses were performed based on the proportional hazard assumption that the relative hazard remains constant over time with different covariate levels (Kuitunen et al., 2021). We also performed landmark survival analysis at 1-year, as described previously (McNamara et al., 2020). The association between variables and overall survival was evaluated using Cox regression. The variables used for signature-based clustering (UV-low vs UV-high), age (continuous), sex (male vs female), stage (I/II vs III/IV), and mutation class (*BRAF* hotspot vs non-*BRAF*) were used to adjust the estimates for the multivariate Cox proportional hazards regression model. The Cox regression results were reported in terms of unadjusted and adjusted hazard ratios (HRs), 95% confidence intervals (CIs) and *p*-values. Details are available in the Supplementary Methods.

### Somatic mutation analysis

Mutational profiles of known driver genes of CM (Cancer Genome Atlas Network, 2015; Hayward et al., 2017) were analyzed and visualized using the Maftools package (Mayakonda et al., 2018). Each sample was classified into *BRAF* hotspot, *RAS* hotspot, *NF1*, and triple wild-type (Triple-WT; CMs without *BRAF* hotspots, *RAS* hotspots, and *NF1* mutations), as described elsewhere (Cancer Genome Atlas Network, 2015).

### Copy number alteration analysis

Somatic copy number alterations (CNAs) were analyzed using raw sequence reads (BAM files). The CNAs of each sample were defined using the ngCGH module and SNPRank segmentation statistical algorithm in Nexus Copy Number 10.0 (BioDiscovery, El Segundo, CA). Segments were classified as copy number gains and losses when the log2 ratio was >0.2 and < -0.2, respectively. Genome-wide frequencies of CNA were visualized using the Copynumber package (Nilsen et al., 2012). CNA regions with statistical differences between the two groups (Chi-square test, *p* < 0.001) were analyzed using the CNVruler software (Kim et al., 2012). Details are provided in the Supplementary Methods.

### Differentially expressed gene and gene set enrichment analysis

RNA sequencing data were downloaded (http://firebrowse.org) and used for differentially expressed gene (DEG) analysis. DEG analysis was performed using “glmQLFit” and “glmQLFTest” in the edgeR package (Robinson et al., 2010). DEGs were defined using a cutoff of log fold change >2 and a q-value less than 0.01. Gene set enrichment analysis was performed using normalized expression data, and gene sets with *p* < 0.01 were considered significantly enriched pathways.

### Tumor purity and immunoprofiling

Curated data of tumor purity, tumor immunity-related features including leukocyte cell fraction, richness of T cell receptors (TCR), and fractions of tumor-infiltrating lymphocytes from TCGA cohort were obtained (Aran et al., 2015; Saltz et al., 2018; Thorsson et al., 2018). The CYT score representing the activity of immune cytolytic effectors was calculated as the geometric mean of normalized RSEM expression of *GZMA* and *PRF1*, as previously described (Rooney et al., 2015). The absolute abundance of 22 immune cell types was inferred from the normalized RNA expression using the CIBERSORTx package (Newman et al., 2019).

### Independent panel sequencing cohort analysis and *in silico* panel simulation

Independent panel sequencing data (“MSK cohort,” cases sequenced using a 468-gene panel covering a 1.2 Mb capture region [MSK-IMPACT468], *N* = 245) were obtained and analyzed from The AACR GENIE project (AACR Project GENIE Consortium, 2017). *In silico* panel simulation was generated from TCGA data using the same region of the panel used in the MSK cohort. Details are provided in the Supplementary Methods.

### Other statistical analyses

Statistical analysis was performed by two-sided Mann–Whitney *U* test for continuous variables and two-sided Chi-square test for categorical variables, and *p* < 0.05 was considered to be statistically significant. All statistical analyses were performed using R software (version 4.1.1). The Mann–Whitney U and Chi-square tests were performed using the ‘wilcox.test’ and ‘chisq.test’ functions from the ‘stats’ R package, respectively.

## Results

### *De novo* mutational signature extraction and unsupervised clustering

**FIGURE 1:**
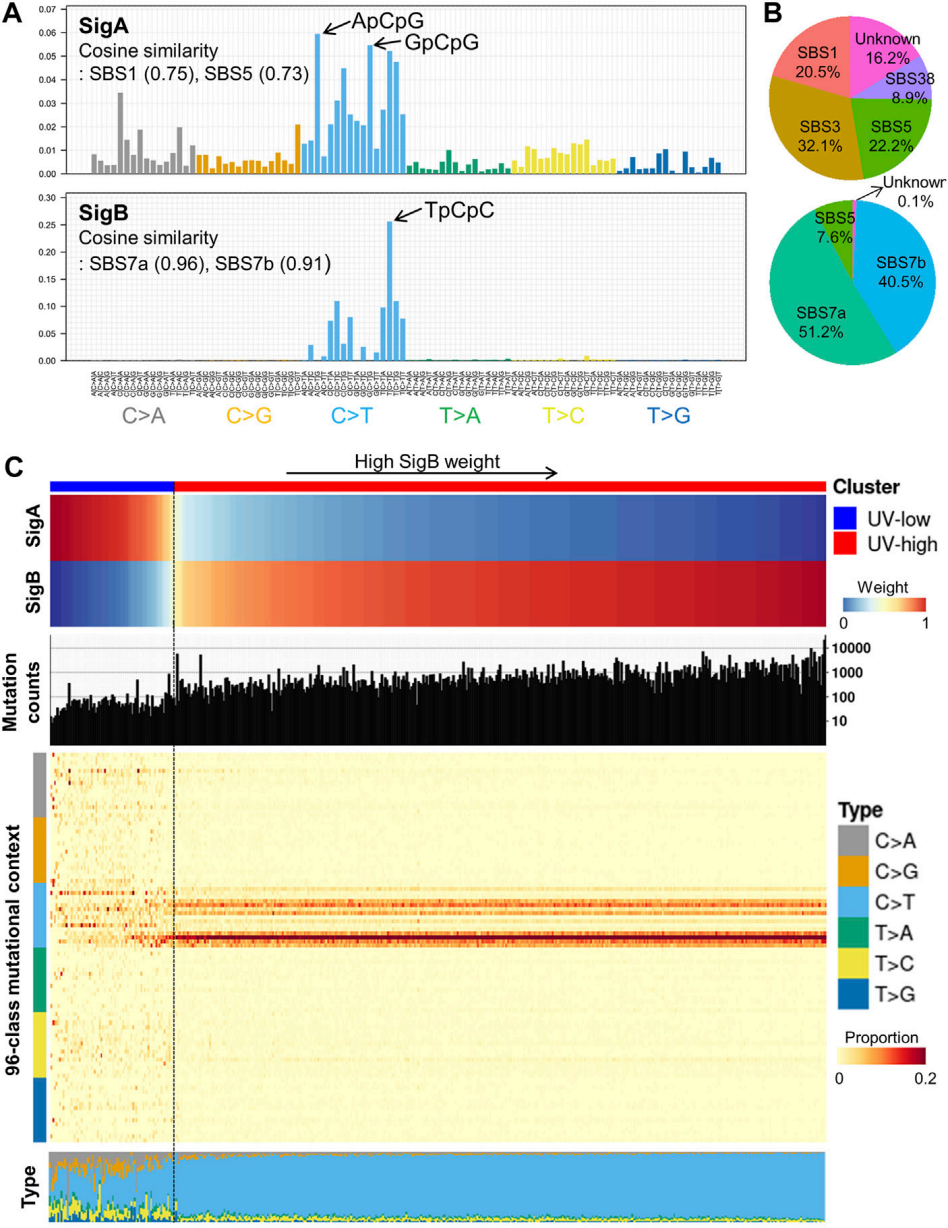
*De novo* mutational signature extraction and unsupervised clustering in TCGA CM dataset. **(A)** *De novo* mutational signature extraction using NMF identified two mutational signatures: “SigA” and “SigB.” The upper and lower bar plots represent the mutational context of SigA and SigB, respectively. **(B)** Pie charts represent signature refitting analysis of SigA and SigB signatures. **(C)** Weight of SigA and SigB, mutational counts, and mutational contexts of TCGA dataset are shown for each sample (*X*-axis). Unsupervised k-means clustering of melanoma samples yielded two clusters: UV-low (*N* = 75) and UV-high (*N* = 391). Samples (column) are arranged by the weight of SigB, and column bar indicates clusters.

From the TCGA CM WES dataset (*N* = 466), we performed *de novo* mutational signature extraction using NMF and found two mutational signatures, “SigA” and “SigB” (Figure 1A; Supplementary Figure S1). SigA showed the highest weight of C > T in the ApCpG and GpCpG contexts and also displayed high similarity to the COSMIC SBS1 (cosine similarity 0.75) and SBS5 (0.73) signatures. SigB showed the highest weight in the TpCpC context and exhibited very high similarity to SBS7a (0.96) and SBS7b (0.91), suggesting a UV signature. As an alternative way to interpret the extracted signatures, signature refitting by known COSMIC signatures of CM revealed SBS1/3/5/38 in SigA and SBS7a/7b/5 in SigB (Figure 1B).

Unsupervised clustering of the TCGA CM dataset by weight of extracted signatures revealed two clusters: a “UV-low” cluster with a dominant SigA signature (*N* = 75), and a “UV-high” cluster with a dominant SigB signature (*N* = 391) (Figure 1C). The UV-high and UV-low clusters showed distinct mutational counts and contexts: a low mutational burden and diffuse distribution of all contexts in the UV-low cluster; in contrast, a high mutational burden and dominant C > T context were observed in the UV-high cluster (Figure 1C). The average mutational context in the UV-low and UV-high clusters showed a high similarity to SigA (cosine similarity 0.88) and SigB (1.00), respectively (Supplementary Figure S2). The two clusters were not affected by the variant calling method, average read depth, history of neoadjuvant chemotherapy, or primary tumor diagnosis (Supplementary Figures S3 and S4).

### Validation of mutational signatures in independent datasets

To validate the mutational signatures extracted from the TCGA data, we performed the same *de novo* mutational signature extraction from two independent datasets. The first dataset included two studies from the ICGC consortium (ICGC dataset, *N* = 235) (Hayward et al., 2017; Campbell, 2020) and the second dataset included seven studies from the SRA database (SRA dataset, *N* = 292) (Krauthammer et al., 2012; Snyder et al., 2014; Hugo et al., 2016; Liang et al., 2017; Riaz et al., 2017; Roh et al., 2017; Shain et al., 2018) (Supplementary Table S1). Mutational signatures extracted from the ICGC and SRA datasets showed high similarity to those from the TCGA cohort: cosine similarity with SigA (ICGC, 0.96; SRA, 0.91) and SigB (ICGC, 1.00; SRA, 0.99) (Supplementary Figure S5; Supplementary Table S2). Consistent with the results obtained from TCGA cohort, two clusters with distinct mutational counts and contexts were drawn by unsupervised clustering in both datasets (Supplementary Figure S5). Mutational counts were significantly higher in the UV-high cluster than in the UV-low cluster in all three datasets (*p* < 2.2e-16) (Supplementary Figure S6). Principal component analysis of the mutational context consistently revealed segregated distributions of UV-low and UV-high clusters in all three datasets (Supplementary Figure S7). In addition, according to a whole-genome sequencing dataset (MELA-AU study in the ICGC dataset), a comparison of mutational contexts using all variants and variants in the exonic region (*in silico* downsampled) showed high similarity (median cosine similarity 0.977), which ensured the reliability of mutational signatures extracted from WES datasets (Supplementary Figure S8).

### Signature refitting analysis

**FIGURE 2:**
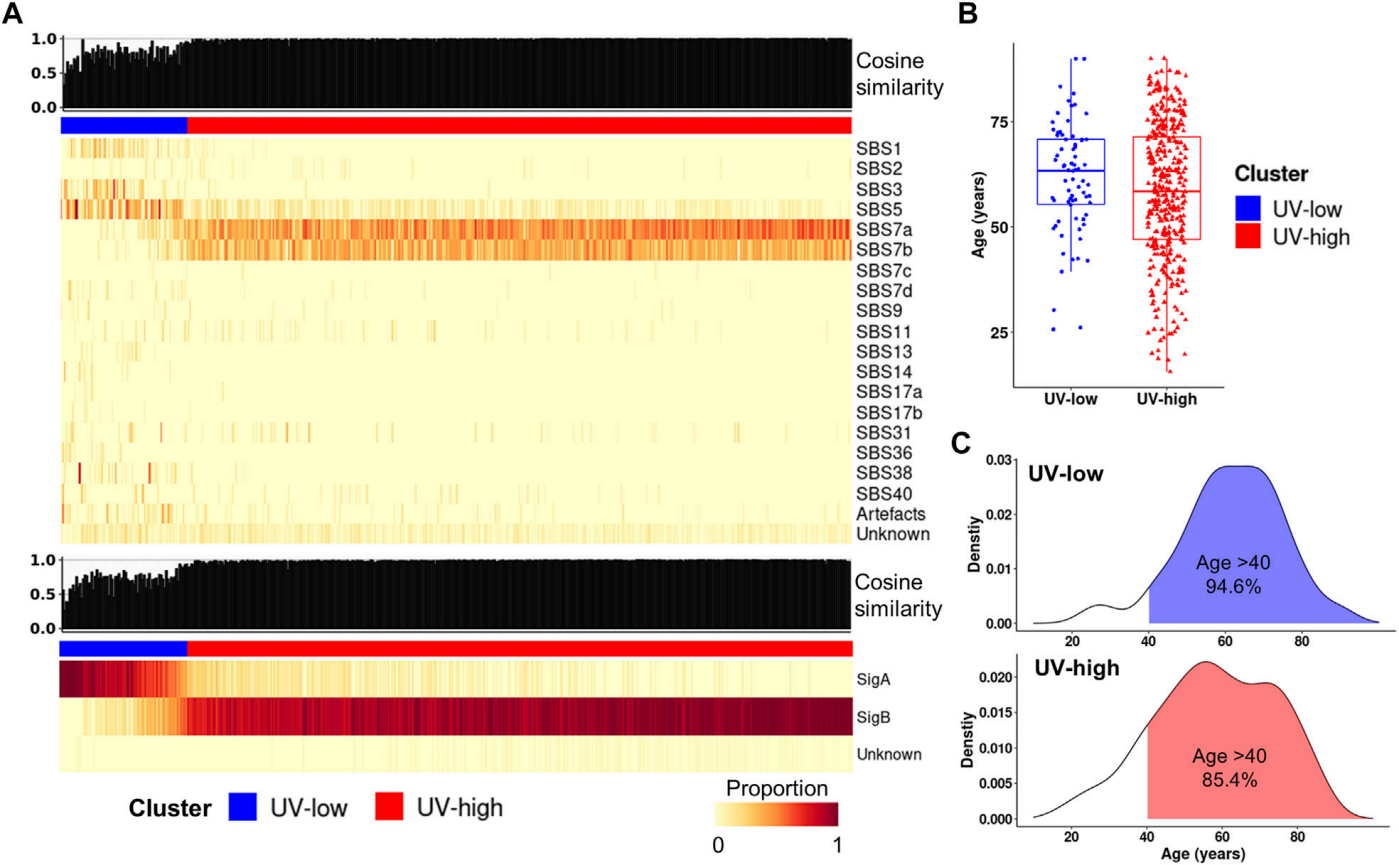
Signature refitting analysis. **(A)** Signature refitting analysis of the TCGA cohort by known COSMIC signatures (top) and extracted signatures (bottom). Samples (column) are arranged by the weight of SigB, and column bar indicates clusters (Same order as Figure 1B). **(B)** Boxplot for age between clusters. **(C)** Density plot showing age distribution for clusters in TCGA cohort (blue, UV-low; red, UV-high).

To compare signature decomposition with known signatures, signature refitting analysis was performed using known mutational signatures of CM in the COSMIC database (Alexandrov et al., 2020) and the extracted signatures (SigA/SigB). Of the known COSMIC signatures, the UV-low cluster displayed a higher weight of age-related signatures (SBS1 and 5), whereas the UV-high cluster exhibited a higher weight of UV signatures (SBS7a and 7b) (Figure 2A). Accordingly, the median age of patients in the UV-low cluster was higher than that of the UV-high cluster, although the difference was not statistically significant (*p* = 0.098) (Figure 2B), and the proportion of patients aged >40 years was higher in the UV-low cluster (94.6%) than in the UV-high cluster (85.4%) (*p* = 0.051) (Figure 2C). As expected, the UV-low and UV-high clusters showed high SigA and SigB weights, respectively (Figure 2A). Consistent with the above findings, the signature refitting analysis of the ICGC and SRA datasets showed results similar to those of TCGA dataset (Supplementary Figure S9).

### Clinical implications of mutational signatures

**FIGURE 3:**
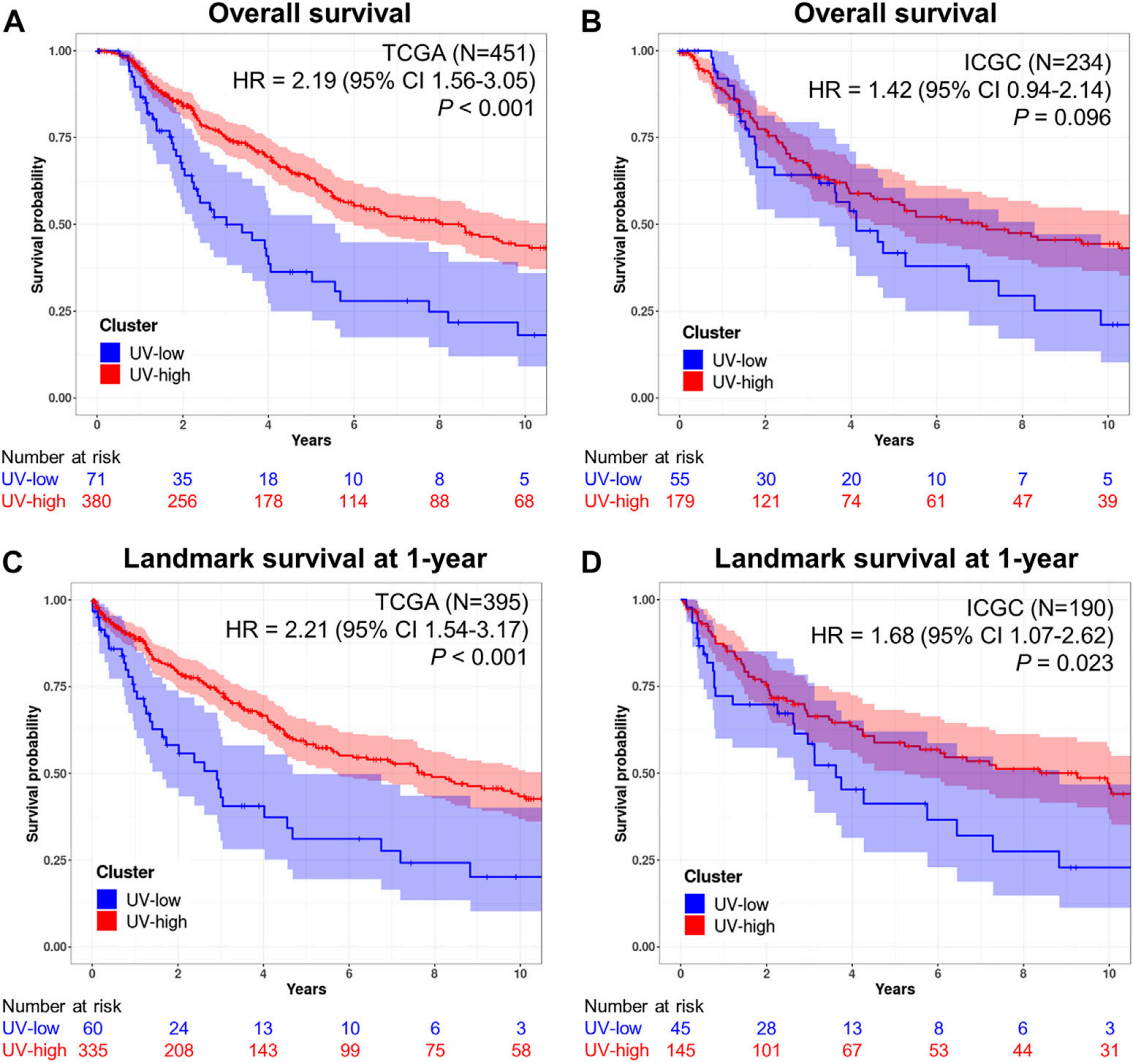
Prognostic implications of mutational signatures. **(A)** Kaplan–Meier curves for overall survival of UV-low (blue) and UV-high (red) clusters in the TCGA dataset (*N* = 451). **(B)** Kaplan–Meier curves for overall survival of UV-low (blue) and UV-high (red) clusters in the ICGC dataset (*N* = 234). **(C)** Kaplan–Meier curves for landmark survival at 1-year of UV-low (blue) and UV-high (red) clusters in the TCGA dataset (*N* = 395). **(D)** Kaplan–Meier curves for landmark survival at 1-year of UV-low (blue) and UV-high (red) clusters in the ICGC dataset (*N* = 190). The shaded areas correspond to 95% CIs. *p*-value was calculated using a two-tailed log-rank test. The numbers beneath each chart indicate the number of patients at risk at each time point.

Here, we investigated the prognostic implications of mutational signature-based clustering. When we compared the overall survival between the two clusters in the TCGA cohort (*N* = 451), the UV-low cluster showed significantly worse overall survival than the UV-high cluster (HR = 2.19, 95% CI 1.56–3.05, *p* < 0.001) (Figure 3A). A poorer prognosis of the UV-low cluster was consistently observed in the ICGC dataset (*N* = 234), but the difference was not statistically significant (HR = 1.42, 95% CI 0.94–2.14, *p* = 0.096) (Figure 3B). The UV-low cluster showed significantly worse landmark survival at 1-year than the UV-high cluster, in both the TCGA cohort (HR = 2.21, 95% CI 1.54–3.17, *p* < 0.001) (Figure 3C) and the ICGC dataset (HR = 1.68, 95% CI 1.07–2.62, *p* = 0.023) (Figure 3D). Age, stage III/IV, and non-*BRAF* mutations were also found to be significant factors for worse overall survival and landmark survival at 1-year in the univariate analysis (Table 1). In multivariate analysis adjusted for age, sex, stage, and mutation class in the TCGA cohort, the UV-low cluster remained the most significant prognostic factor for overall survival (HR = 2.14, 95% CI 1.47–3.11, *p* < 0.001) (Table 1). UV-low cluster also remained the most significant prognostic factor for landmark survival at 1-year (HR = 2.07, 95% CI 1.37–3.11, *p* < 0.001) (Table 1). Age and advanced stage (III/IV) remained significant prognostic factors in the multivariate analysis. In the subgroup analysis, the UV-low cluster correlated with significantly poorer overall survival than the UV-high cluster in all subgroups except *BRAF* hotspot mutation status: stage I/II (*p* < 0.001), stage III/IV (*p* = 0.011), age >40 (*p* < 0.001), and non-*BRAF* hotspot (*p* = 0.001) (Supplementary Figure S10). Even in the *BRAF* hotspot mutation subgroup, the UV-low cluster displayed a trend of poorer overall survival (*p* = 0.059). The proportions of advanced stage (stage III/IV) (*p* = 0.024), and greater invasion depth (Clark IV/V) (*p* = 0.001), ulceration (*p* = 0.002) were also significantly higher in the UV-low cluster (Supplementary Figure S10).

**TABLE 1:** Multivariate survival analysis and landmark survival at 1-year of cutaneous melanomas in TCGA.

Variables	Overall survival unadjusted HR (95% CI; *p-*value)	Overall survival adjusted HR (95% CI; *p-*value^a^)	Landmark survival (1-year) unadjusted HR (95% CI; *p-*value)	Landmark survival (1-year) adjusted HR (95% CI; *p-*value^a^)
Cluster				
UV-high	Reference	Reference	Reference	Reference
UV-low	2.19 (1.56–3.05; *p* < 0.001)	2.14 (1.47–3.11; *p* < 0.001)	2.21 (1.54–3.17; *p* < 0.001)	2.07 (1.37–3.11; *p* < 0.001)
Age				
(Continuous)	1.02 (1.02–1.03; *p* < 0.001)	1.02 (1.01–1.03; *p* < 0.001)	1.03 (1.02–1.04; *p* < 0.001)	1.02 (1.01–1.04; *p* < 0.001)
Sex				
Female	Reference	Reference	Reference	Reference
Male	1.14 (0.86–1.51; *p* = 0.370)	1.10 (0.81–1.48; *p* = 0.547)	1.11 (0.82–1.50; *p* = 0.492)	1.03 (0.75–1.42; *p* = 0.863)
Clinical stage				
Stage I/II	Reference	Reference	Reference	Reference
Stage III/IV	1.70 (1.28–2.27; *p* < 0.001)	1.65 (1.23–2.21; *p* < 0.001)	1.54 (1.13–2.10; *p* = 0.006)	1.51 (1.10–2.06; *p* = 0.010)
Mutation type				
*BRAF* hotspot	Reference	Reference	Reference	Reference
Non-*BRAF*	1.44 (1.09–1.90; *p* = 0.010)	0.97 (0.71–1.32; *p* = 0.837)	1.57 (1.17–2.11; *p* = 0.003)	1.04 (0.74–1.45; *p* = 0.840)

HR, hazard ratio; CI, confidence interval. *p*-values were calculated using two-sided log-rank tests.

a Multivariate analysis was performed using an adjusted multivariate Cox proportional hazards regression model including cluster (UV-low vs. UV-high), age (continuous), sex (male vs. female), stage (I/II vs. III/IV), and mutation class (*BRAF* hotspot vs. non-*BRAF*).

### Genomic and functional characteristics

**FIGURE 4:**
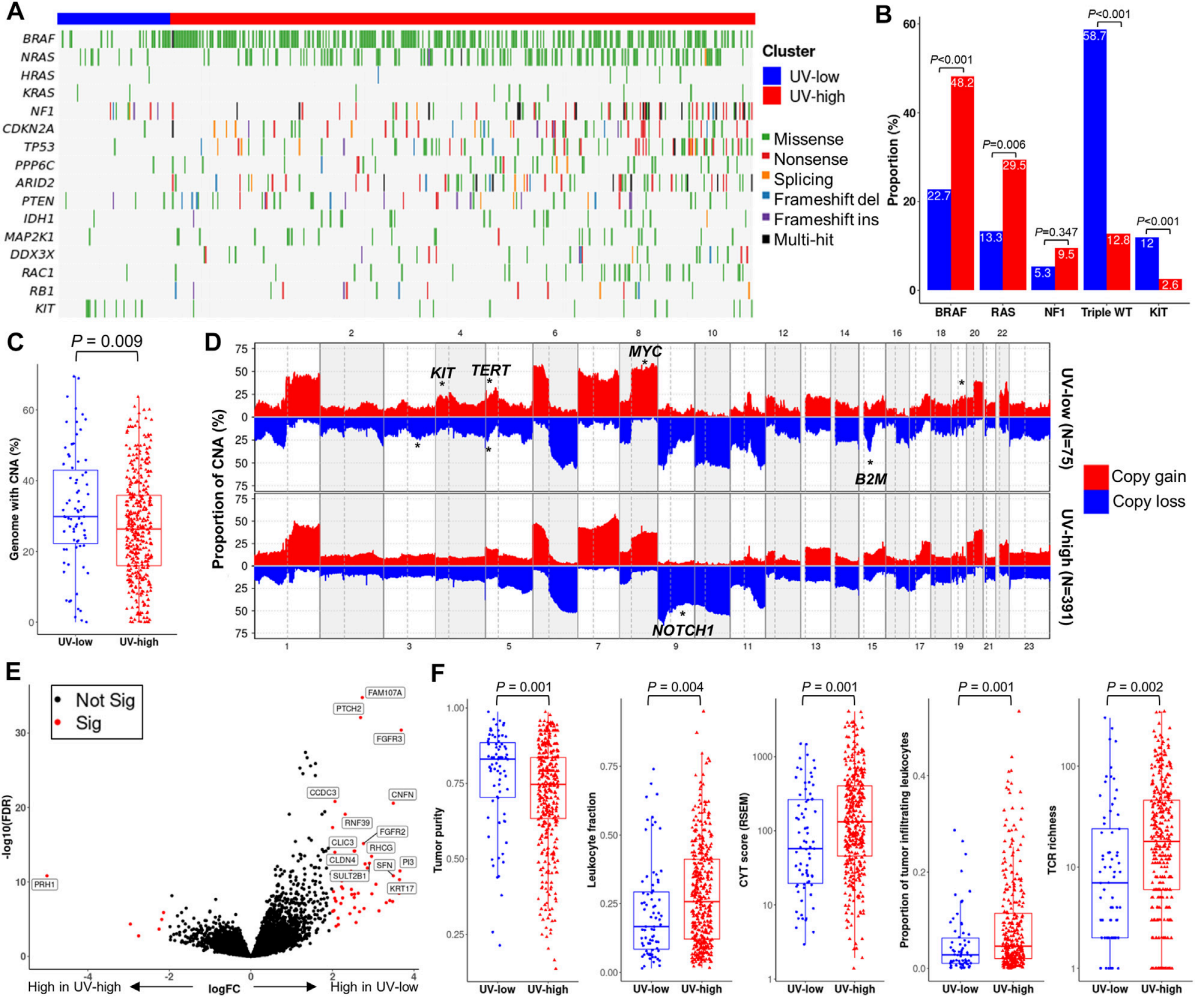
Genomic and functional characterization. **(A)** Mutational landscape of known driver genes of melanoma. Samples (column) are arranged by the weight of SigB (Same as Figure 1B). **(B)** Proportion of mutational class (*BRAF* hotspot, *RAS* hotspot, *NF1*, triple wild type (WT)) and *KIT* non-silent mutations. *p*-value was calculated using a two-sided chi-square test. **(C)** Boxplot showing the proportion of genomes with copy number alteration (CNA). *p*-value was calculated using a two-sided Mann-Whitney *U* test. **(D)** Genome-wide CNA plot of each cluster. CNA regions with statistically significant enrichment (chi-square test *p* < 0.001) are marked with asterisks on putative driver genes. **(E)** Volcano plot with *x*-axis (log fold change) and *y*-axis (- log false discovery rate (FDR)), in which genes are colored by significance (FDR <0.01) (red, significant; black, non-significant). Representative differentially expressed genes are marked with gene names. **(F)** Boxplot of tumor purity and representative parameters of tumor immunity. *p*-value was calculated using a two-sided Mann-Whitney *U* test.

We further compared the genomic and functional characteristics of the UV-low and UV-high clusters. Non-silent mutations in known driver genes were lower in the UV-low cluster than in the UV-high cluster (Figure 4A). In the context of CM mutational class (Cancer Genome Atlas Network, 2015), the UV-low cluster showed a significantly lower proportion of *BRAF* and *RAS* hotpot mutations, and, correspondingly, a higher proportion of triple-wild-type (triple-WT) than the UV-high cluster (Figure 4B). Among the other driver alterations, only *KIT* non-silent mutations showed a significantly higher proportion in the UV-low cluster. In contrast to the higher mutation burden in the UV-high cluster, the CNA burden (percentage of genome with CNA) of the UV-low cluster was significantly higher than that of the UV-high cluster (*p* = 0.009) (Figure 4C). Of the CNAs, 3q loss, 4p gain (*KIT*), 5p gain (*TERT*), 5p loss, 8q gain (*MYC*), 15q loss (*B2M*), and 9q loss (*NOTCH1*) were significantly different between the two clusters (*p* < 0.001) (Figure 4D; Supplementary Table S5).

DEG analysis revealed 64 significant DEGs between the UV-low and UV-high clusters (q-value < 0.01) (Supplementary Table S6). A number of keratinocyte differentiation-related genes (*CNFN, FGFR2/3, KRT17, PI3, PTCH2,* and *SFN*) were significantly overexpressed, whereas *PRH1* was downregulated in the UV-low cluster (Figure 4E). Pathway analysis showed that keratinocyte differentiation-related pathways were significantly upregulated in the UV-low cluster, whereas immune-related pathways were upregulated in the UV-high cluster (Supplementary Table S7). Notably, tumor purity was significantly higher in the UV-low cluster (*p* = 0.001), whereas cancer immunity-related characteristics, such as leukocyte fraction (*p* = 0.004), proportion of tumor-infiltrating leukocytes (*p* = 0.001), CYT score (Rooney et al., 2015) (*p* = 0.001), and T cell receptor (TCR) richness (*p* = 0.002) were significantly lower in the UV-low cluster (Figure 4F). Accordingly, *in silico* immunoprofiling analysis revealed that the UV-low cluster had a lower fraction of immune cells (*p* = 0.007). In addition, the fractions of immune cell subtypes related to tumor immunity, including M1 macrophages, CD4^+^ memory activated T cells, regulatory T cells, and CD8^+^ T cells, were significantly higher in the UV-high cluster (Supplementary Figure S11).

We also compared our signature-based clustering with known subtypes from TCGA study (UV, mutation, RNA, miRNA, methylation, and protein subtypes). As expected, the UV-low and UV-high clusters displayed an almost exclusive ratio of “Not UV” and “UV” (UV subtypes), respectively. Furthermore, while the UV-low cluster exhibited a significantly higher proportion of triple-WT and “Keratin” expression subtypes, the UV-high cluster comprised significantly higher proportion of *BRAF* and *RAS* subtypes (mutation) (Supplementary Figure S12). The UV-high cluster also exhibited a significantly higher proportion of “Immune” expression and hypo-methylated subtypes (*p* < 0.01) (Supplementary Figure S12).

### Independent panel sequencing cohort analysis and *in silico* panel simulation

**FIGURE 5:**
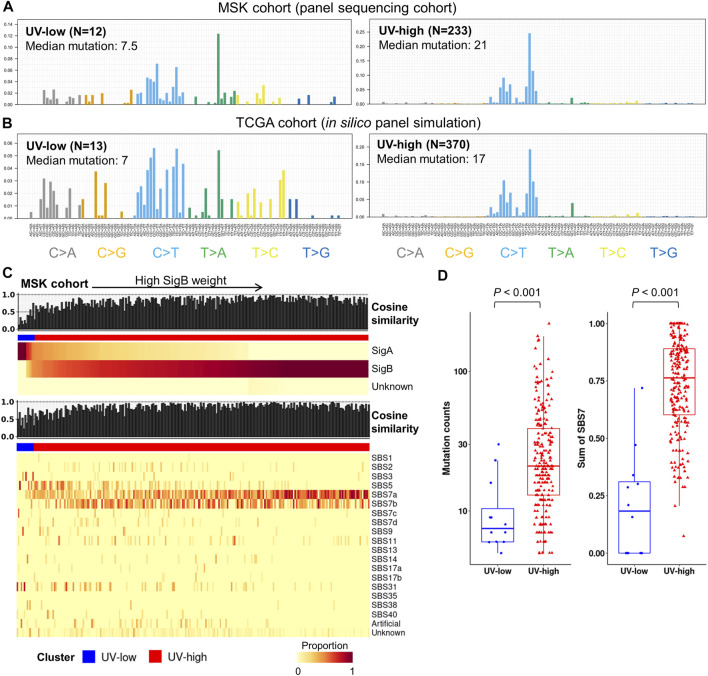
Independent panel sequencing cohort analysis and *in silico* panel simulation of TCGA cohort. **(A)** Average mutational context in independent panel sequencing cohort (MSK cohort), in which samples are classified by dominant signatures. **(B)** Average mutational context of UV-low and UV-high clusters from *in silico* panel simulation of TCGA cohort. Samples with less than five single nucleotide variants of simulated data were excluded. **(C)** Signature decomposition analysis of MSK cohort by extracted signatures (top) and known COSMIC signatures (bottom). Samples (column) were arranged by the weight of SigB, and column bar indicates clusters. **(D)** Box plots showing mutation counts and SBS7 signatures (SBS7a/7b/7c/7d) for each cluster in MSK cohort. *p*-value was calculated using a two-sided Mann-Whitney *U* test.

To investigate the feasibility of mutational signature-based classification of panel sequencing data, we analyzed independent panel sequencing cohort data (“MSK cohort”, *N* = 245) (AACR Project GENIE Consortium, 2017). Each sample was divided into UV-high (SigB weight >0.5) and UV-low (SigB weight <0.5) clusters by signature refitting analysis. The UV-high and UV-low clusters in the MSK cohort showed distinct average mutational contexts (Figure 5A). In addition, we performed *in silico* panel simulation of the TCGA dataset using the same target region of the MSK cohort and compared the results before and after panel simulation. Although mutation counts were reduced to a median of 2.96% of those from WES, the mutational context was largely coherent with a median cosine similarity of 0.81 before and after panel simulation (Supplementary Figure S13). UV-low and UV-high clusters from the panel simulation of TCGA cohort showed distinct average mutational contexts (Figure 5B), which closely resembled the MSK cohort. According to the signature refitting analysis of the MSK cohort, most samples in the UV-high cluster showed known UV signatures (SBS7a/b/c/d) with high cosine similarity (Figure 5C). The UV-high cluster showed significantly higher mutational counts than the UV-low cluster did (*p* < 0.001) (Figure 5D). The UV-high cluster showed a significantly higher weight of UV signatures (SBS7a/b/c/d) (*p* < 0.001) (Figure 5E) and dominant C > T mutational contexts (Supplementary Figure S14).

## Discussion

The mutational characteristics and clinico-genomic features of CMs with high UV signatures, including high mutational burden, predominant UV signatures, and favorable survival, have been adequately described (Hayward et al., 2017; Trucco et al., 2019). However, the nature of these features in CMs with low UV signatures is still largely unknown. In this study, we investigated the mutational signatures of CMs from TCGA dataset and their clinico-genomic associations. Based on the mutational signatures, CMs were grouped into two clusters with distinct clinico-genomic and functional characteristics: UV-high and UV-low clusters. CMs belonging to the UV-low cluster were associated with a low mutational burden, a mutational signature with high similarity to SBS1/5 signatures, and worse overall survival than the UV-high cluster. We further revealed that UV-high and UV-low clusters can be distinguished using panel sequencing data, which are commonly generated in routine clinical practice.

From TCGA CM dataset, two distinct mutational signatures (SigA and SigB) were identified in this study. The major signature SigB showed a predominant C > T in the TpCpC context and very high similarity to the COSMIC SBS7a and SBS7b signatures, suggesting that this is a typical UV signature (Alexandrov et al., 2020). In contrast to SigB, SigA showed a high weight of C > T in the ApCpG and GpCpG contexts, and a high similarity to the COSMIC SBS1/5 signatures. These two distinct signatures were consistently defined in two independent datasets (ICGC and SRA), indicating the reliability of the two signatures identified in this study. As the evidence of homologous recombination deficiency in CMs is scarce (Nguyen et al., 2020), the SBS3 component in Sig A is likely due to the difficulty of distinguishing between the SBS3 and SBS5 signatures (Koh et al., 2021). Sig A also displayed a minor proportion of the SBS38 signature, which is associated with a proposed etiology of indirect damage from UV (Alexandrov et al., 2020). Mutational signature-based unsupervised clustering of CMs in the three datasets revealed two CM clusters. The major cluster (79%) harbored an almost exclusive UV signature (SigB), whereas the other cluster (21%) showed little or no UV signature (SigA). Therefore, we named the major cluster the “UV-high cluster” and the minor cluster the “UV-low cluster”. Interestingly, CMs belonging to the UV-low cluster showed significantly worse overall survival than those belonging to the UV-high cluster. This finding was consistently observed in the ICGC dataset, suggesting reasonable validity. In addition, the UV-low cluster remained an independent prognostic factor in multivariate analysis, together with traditional prognostic factors, such as age, tumor stage, and mutational type (*BRAF* hotspot or not). Among these factors, the UV-low cluster was the most significant, suggesting that the UV signature may play a fundamental role in the prognosis of CM. There are only a few known clinico-genomic factors predicting prognosis in CM (Gerami et al., 2015; Cancer Genome Atlas Network, 2015; Garg et al., 2021). Our data are consistent with those of previous studies that demonstrated the prognostic implication of the UV signature (Trucco et al., 2019; Pham et al., 2020; Vicente et al., 2022). One of the characteristics of the UV-low cluster is lower mutational and higher CNA burdens, which is similar to the genomic profiles of acral melanoma, a rare subtype of melanoma that has a poorer prognosis than CM (Liang et al., 2017; Rabbie et al., 2019; Newell et al., 2020). Interestingly, the key genomic alterations of acral melanomas described in a previous study (Newell et al., 2020) (such as the highest proportion of triple-WT tumors, common *KIT* alterations, and lower frequencies of *BRAF/RAS* hotspot mutations) are consistent with the characteristics of the UV-low cluster in this study. In line with our findings, another recent study reported results from epigenomic mapping which also suggest that UV-low CMs more closely resemble acral melanomas rather than UV-high CMs (Vicente et al., 2022).

In terms of the repertoire of mutational signatures, CM is known to be much simpler than other solid cancers (Kim et al., 2016; Letouzé et al., 2017; Gulhan et al., 2019; Alexandrov et al., 2020). Accordingly, in this study, CMs in the UV-high cluster showed little heterogeneity in terms of mutational context; however, CMs in the UV-low cluster showed a more heterogeneous mutational context, suggesting that the tumorigenesis of CMs in UV-low clusters is diverse. Based on the age distribution and age-related signatures (SBS1/5) of the UV-low cluster, we presumed that aging is one of the major factors involved in the tumorigenesis of CMs in UV-low clusters (Alexandrov et al., 2020). Signature refitting analysis also revealed consistent components in both signatures across the three databases. These data support the two distinct natures of the mutational signatures in CM and also suggest that the effect of other signatures on CM is minimal.

In this study, the UV-low cluster showed a significantly lower leukocyte fraction, proportion of tumor-infiltrating leukocytes, CYT score, and TCR richness than the UV-high cluster. It has been established that low immune cell infiltration is associated with poor clinical outcome (Barnes and Amir, 2017; Thorsson et al., 2018; Li et al., 2021). Therefore, the poorer survival in the UV-low cluster may be explained by lower activation of antitumor immunity. Additionally, the UV-low cluster showed significantly higher expression of genes related to keratinocyte differentiation. These results are consistent with the significantly higher proportion of “Keratin” subtypes and significantly lower proportion of “Immune” subtypes found in the UV-low cluster (Cancer Genome Atlas Network, 2015). In our study, the “Hypo-methylated” subtype comprised a significantly higher proportion of the UV-high cluster compared to the UV-low cluster, indicating the possibility of distinct DNA methylation profiles. A previous study suggested that distinct DNA methylation profiles between UV-high and UV-low CMs may play a role in immunomodulation and alteration of immune cell composition (Vicente et al., 2022).

Despite its prognostic implications, mutational signature analysis has not been widely adopted in the clinical management of CMs owing to the difficulty of acquiring reliable signatures in routine clinical settings using panel sequencing (Abbasi and Alexandrov, 2021). Considering this situation, we explored whether panel sequencing data could be used to obtain proper mutational signatures. Using both real-world panel sequencing data and a simulation of panel sequencing from the WES dataset, we revealed that UV-high and UV-low clusters of CMs could be distinguished from panel sequencing data. Recent studies have revealed that the detection of mutational signatures from panel sequencing data is available in patients with breast, ovarian, and lung cancer as well as melanoma, to determine their response to PARP and PD-L1 inhibitors (Gulhan et al., 2019; Färkkilä et al., 2020; Chong et al., 2021). Together with previous studies, our results support the feasibility of mutational signature-based classification using panel sequencing data, which may have potential clinical implications. Further studies are required to verify the sensitivity and specificity of mutational signature-based classification and its clinical implications, such as treatment response and prognostic prediction.

This study had several limitations. First, most of the datasets used in this study were WES datasets. Although WES is less accurate than whole-genome sequencing for obtaining reliable mutational signatures, WES can be broadly applied using many more samples and can recapitulate whole-genome sequencing-based mutational signatures. Second, although we observed consistent mutational contexts from the panel sequencing cohort data, this finding was not validated by an independent panel sequencing dataset due to the lack of a proper dataset for this purpose. Compared to WES data, panel sequencing data usually harbors relatively higher sequencing depth, which can result in varied mutation results due to differential sensitivity in mutation detection. Further studies are required to verify the applicability of panel sequencing data. Third, mutational signatures extracted *in silico* need additional validation to prove their biological implications and whether they are independent of known signatures.

## Conclusion

Our study revealed the mutational signatures of CMs and the mutational signature-based clustering of CM into two clusters with distinct clinico-genomic characteristics. Our results support the clinical application of mutational signatures in the classification of CMs for management and prognosis prediction.

## Data Availability

Publicly available datasets were analyzed in this study. This data can be found here: The data generated in this study are available within the article and its Supplementary Data Files. The raw somatic mutation data from TCGA database analyzed in this study were obtained from https://portal.gdc.cancer.gov. The raw somatic mutation data from the ICGC database analyzed in this study were obtained from https://dcc.icgc.org/. The raw sequencing reads data analyzed in this study were obtained from the Sequence Reads Archive (SRA) at SRP067938, SRP090294, and SRP095809, and from the Database of Genotypes and Phenotypes (dbGaP) at phs000178. v11. p8, phs000933. v2. p1, phs000977. v1. p1, phs001041. v1. p1, phs001036. v1. p1, and phs001425. v1. p1. All codes to reproduce our study results and figures have been deposited with GitHub (https://github.com/Yoon-Seob-Kim/TCGA_melanoma). All other data is available within the Supplementary Data.
