# Supplementary material for: Two subtypes of cutaneous melanoma with distinct mutational signatures and clinico-genomic characteristics
Source: Front Genet. 2022 Sep 29;13:987205. doi: 10.3389/fgene.2022.987205 (PMC9557124; doi:10.3389/fgene.2022.987205)

Supplementary materials

Supplementary Methods

Selection of mutation in exonic region from ICGC dataset

Mutations in exonic region were selected using functional annotation of ICGC mutation calling data. Mutations with "consequence_type" of follows were selected as exonic mutations and used for the analysis. : disruptive_inframe_deletion, disruptive_inframe_insertion, frameshift_variant, inframe_deletion, inframe_insertion, exon_variant, missense_variant, start_lost, stop_gained, stop_lost, stop_retained_variant, stop_lost, stop_retained_variant, synonymous_variant.

Analysis of somatic mutations in SRA dataset

The raw sequence reads were aligned to human genome (GRCh37) using BWA, and somatic mutation calls were made by Mutect2 pipeline of GATK, according to the best practice guideline (v 4.0.3.0, https://software.broadinstitute.org/gatk). Samples without germline control, cell line samples, and samples with history of previous immunotherapy were excluded for the analysis.

Survival analysis

For overall survival analysis and likelihood-ratio tests, the 'Surv' function, 'survfit' function, and 'coxph' function from the 'survival R package was used. For multivariate analysis of an adjusted multivariate Cox proportional hazards regression model used in the survival analysis, the 'analyse_multivariate' function from the 'survivalAnalysis' R package was used. Kaplan–

Meier curves were visualized using ‘ggsurvplot’ function from the ‘survminer’ R package

Statistical difference test of copy number alteration

CNA regions with statistical difference between two clusters (chi-square test $P < 0.001$) were analyzed using “Reciprocal overlap” method of CNVruler software (v1.3.3.2) (Kim et al., 2012), in which integration and identification of common CNV regions with overlap of one CNV with another over a predefined threshold value (0.5). For input CNA segments, segments less than 1Kb were excluded, and for output CNA regions with statistical difference, regions less than 1Mb were excluded.

Known mutational signatures in melanoma

Known mutational signatures of melanoma were obtained (Alexandrov et al., 2020), and were used for the signature refitting: SBS1, SBS2, SBS3, SBS5, SBS7a, SBS7b, SBS7c, SBS7d, SBS9, SBS11, SBS13, SBS14, SBS17a, SBS17b, SBS31, SBS36, SBS38, SBS40, SBS43, SBS45, SBS49, SBS52, SBS55, and SBS58. The weights of artificial signatures (SBS43, SBS45, SBS49, SBS52, SBS55, and SBS58) were summed up and denoted as “Artificial” signature.

Independent panel sequencing cohort analysis and *in silico* panel simulation

For independent panel sequencing cohort analysis, curated mutation calls from independent panel sequencing cohort were downloaded (cases using MSK-IMPACT468 in The AACR GENIE project, release 7.0-public, available at synapse.org ID syn20333031). This cohort was

composed of 245 melanoma patients who underwent hybridization-based targeted panel sequencing of 468 cancer related genes from tumor and germline control. Cases with less than 5 SNVs were excluded for the analysis.

For *in silico* panel simulation, region file of MSK-IMPACT468 was downloaded. For the application of hg38 mutation calls of TCGA samples, lift over of region to hg38 reference genome was done using LiftOver tool (https://genome.ucsc.edu/cgi-bin/hgLiftOver). Tabix function of Samtools was used to filter variants inside the panel among variants of TCGA cohort. Cases with less than 5 SNVs after the simulation were excluded for the analysis.

Reference

Alexandrov LB, Kim J, Haradhvala NJ, Huang MN, Tian Ng AW, Wu Y, et al. The repertoire of mutational signatures in human cancer. Nature 2020;578:94-101.

Kim JH, Hu HJ, Yim SH, Bae JS, Kim SY, Chung YJ. CNVRuler: a copy number variation-based case-control association analysis tool. Bioinformatics 2012;28:1790-2.

Supplementary Figure Legends

Supplementary Figure S1. Metrics for the most appropriate rank of non-negative factorization. Plot shows the average sample cosine distance (red, left y-axis) and the average average silhouette width (blue, right y-axis) according to the number of total signatures calculated by the SigProfilerExtractor tool. Two signatures are suggested as the most appropriate number of total signatures (colored by grey).

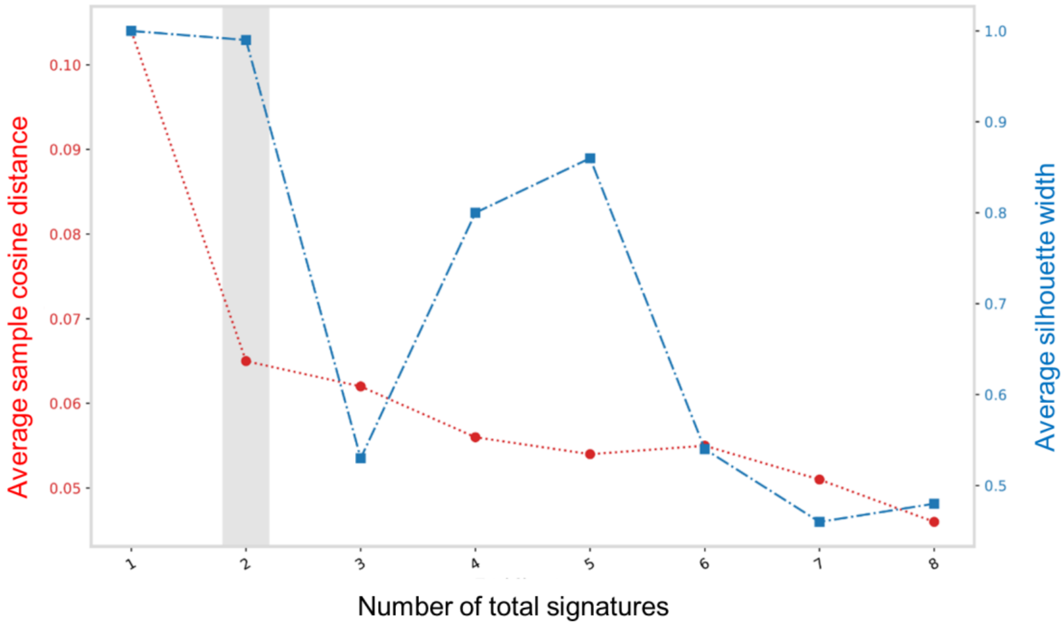

Supplementary Figure S2. Average mutational context of total TCGA cohort, UV-low and UV-high clusters. Distinct average mutational context was observed between UV-low and UV-high cluster.

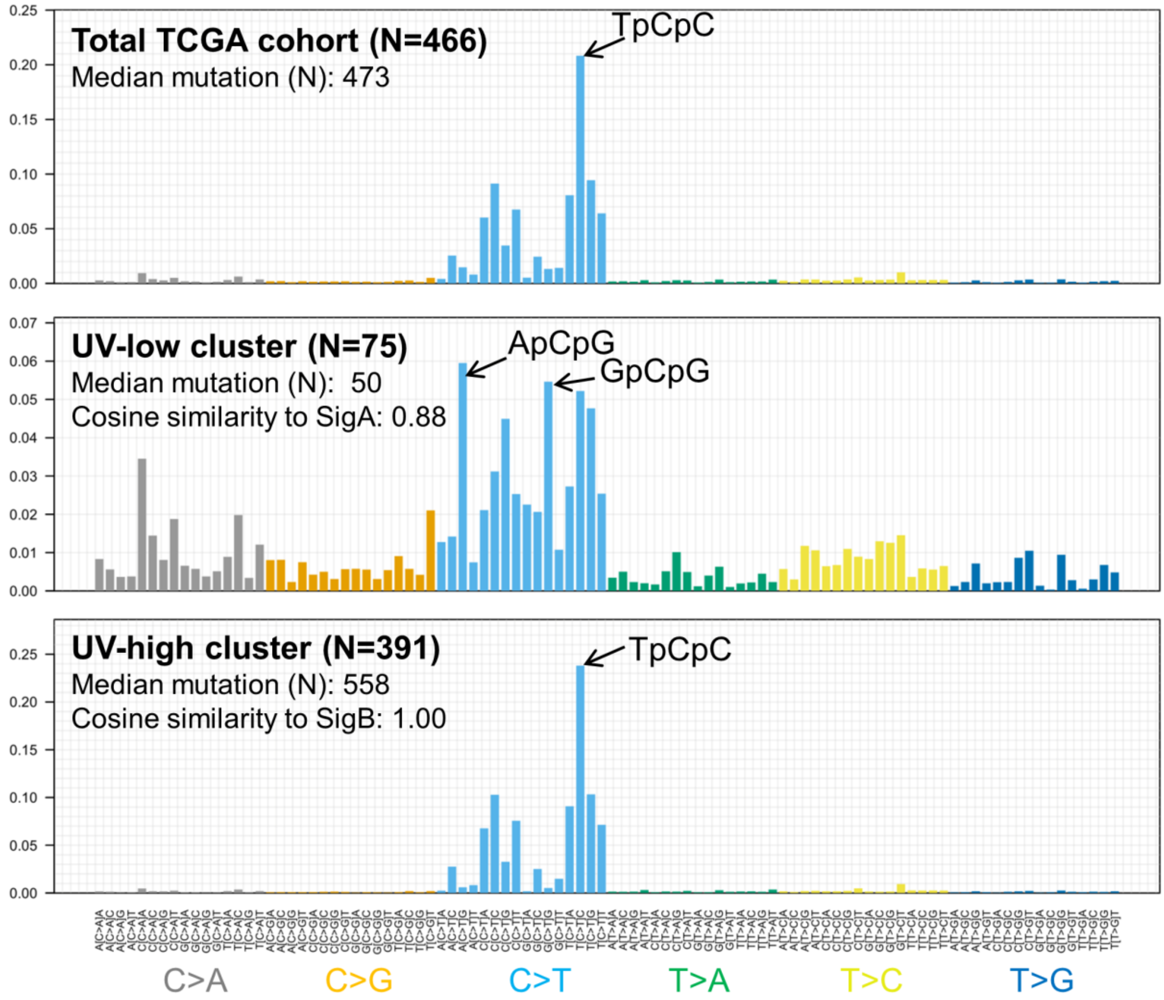

Supplementary Figure S3. Comparison of mutational context using variants called by Mutect2 and Varscan2. High cosine similarity (median cosine similarity 0.998) were found between mutational context using variants called by pipeline used in this study (Mutect2) and another pipeline (Varscan2). Samples (column) were arranged by the weight of SigB (Same order as **Fig. 1b**).

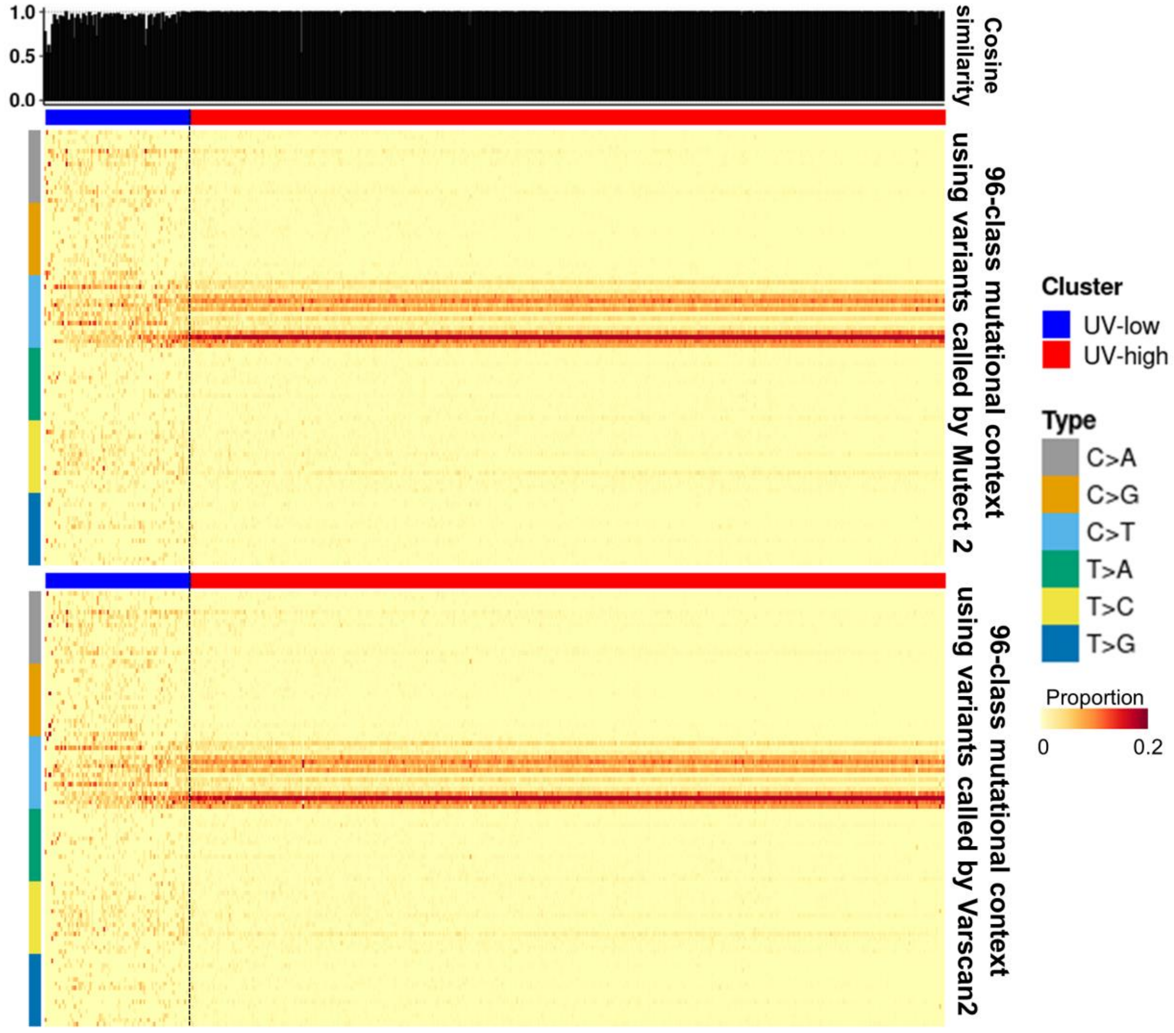

Supplementary Figure S4. Comparison of average read depth, proportion of neoadjuvant chemotherapy, and primary tumor diagnosis between UV-low and UV-high clusters. There was no significant difference of average read depth of exonic region from ENSEMBL (*P* = 0.740), proportion of history of neoadjuvant chemotherapy (*P* = 0.409), and primary tumor diagnosis (*P* = 0.656) between UV-low and UV-high clusters. *P*-value was calculated using a two-sided Mann-Whitney U test (read depth) or two-sided chi-square test (neoadjuvant chemotherapy and primary tumor).

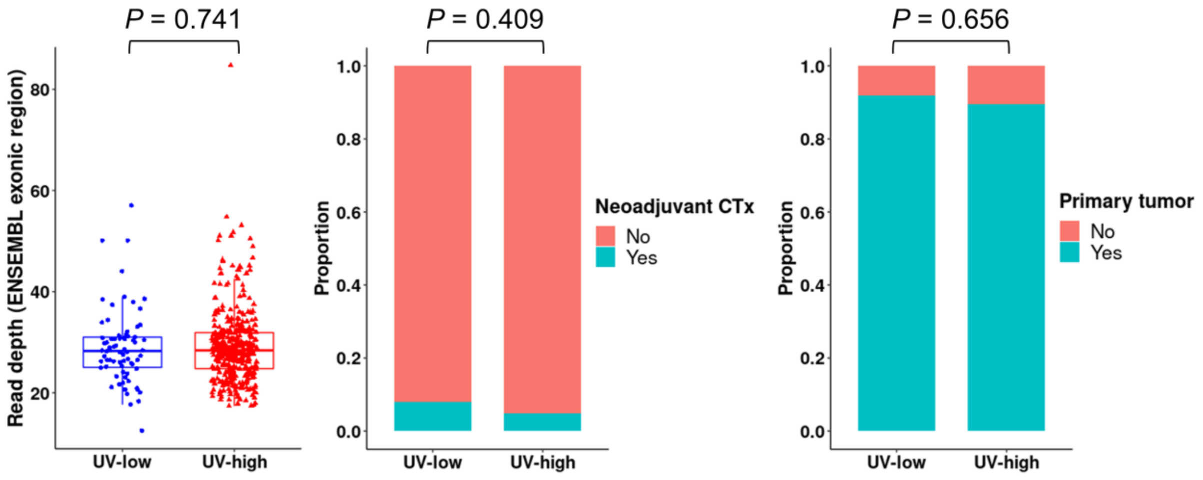

Supplementary Figure S5. NMF-based unsupervised clustering of mutational signatures in ICGC and SRA datasets. Mutational signatures extracted from (A) ICGC and (B) SRA datasets showed high similarity to those from TCGA cohort: cosine similarity of SigA (ICGC, 0.96; SRA, 0.91) and SigB (ICGC, 1.00; SRA, 0.99). Unsupervised clustering found two clusters with distinct mutational counts and context in ICGC and SRA datasets, respectively. Samples (column) were arranged by the weight of SigB of each dataset.

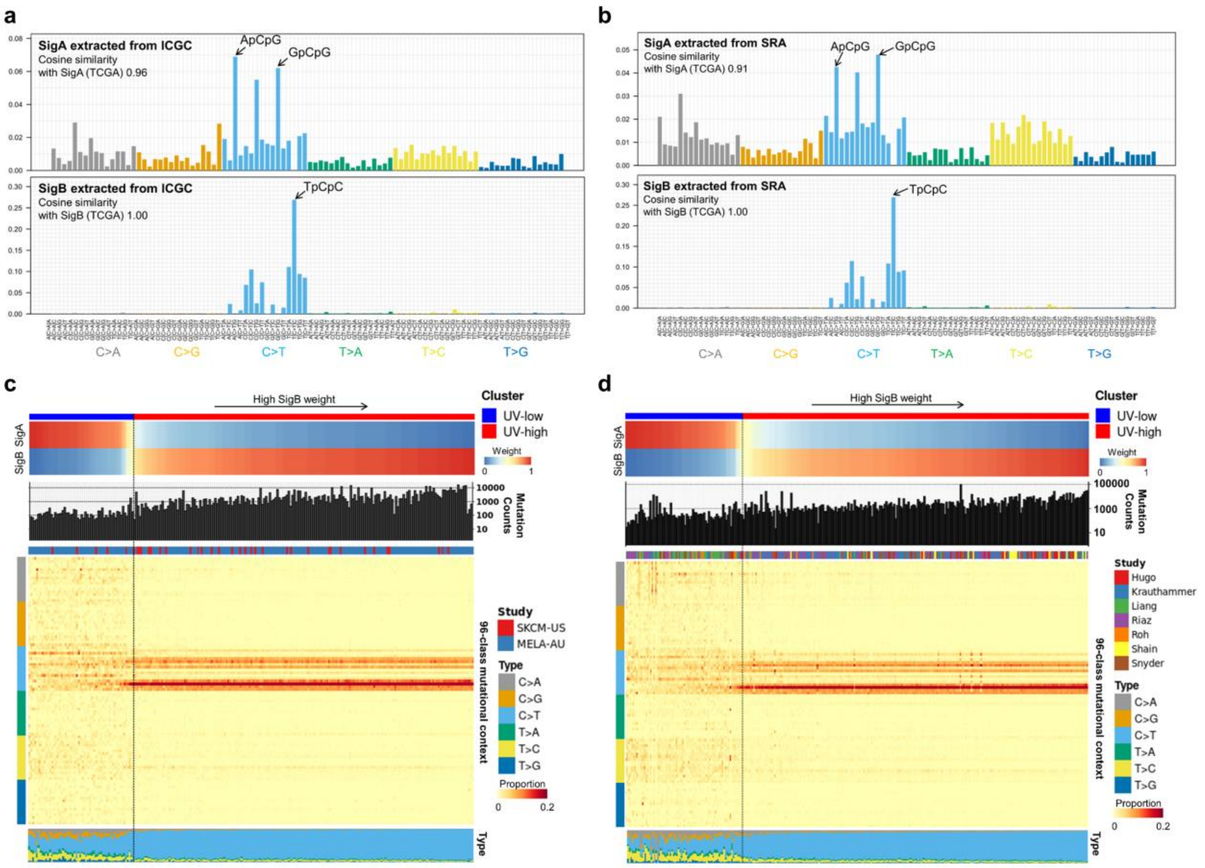

Supplementary Figure S6. Comparison on mutation counts in each dataset (TCGA, ICGC, and SRA). Mutational counts were significantly higher in UV-high cluster compared to UV-low cluster in TCGA, ICGC, and SRA datasets (*P* < 2.2e-16). *P*-value was calculated using a two-sided Mann-Whitney U test.

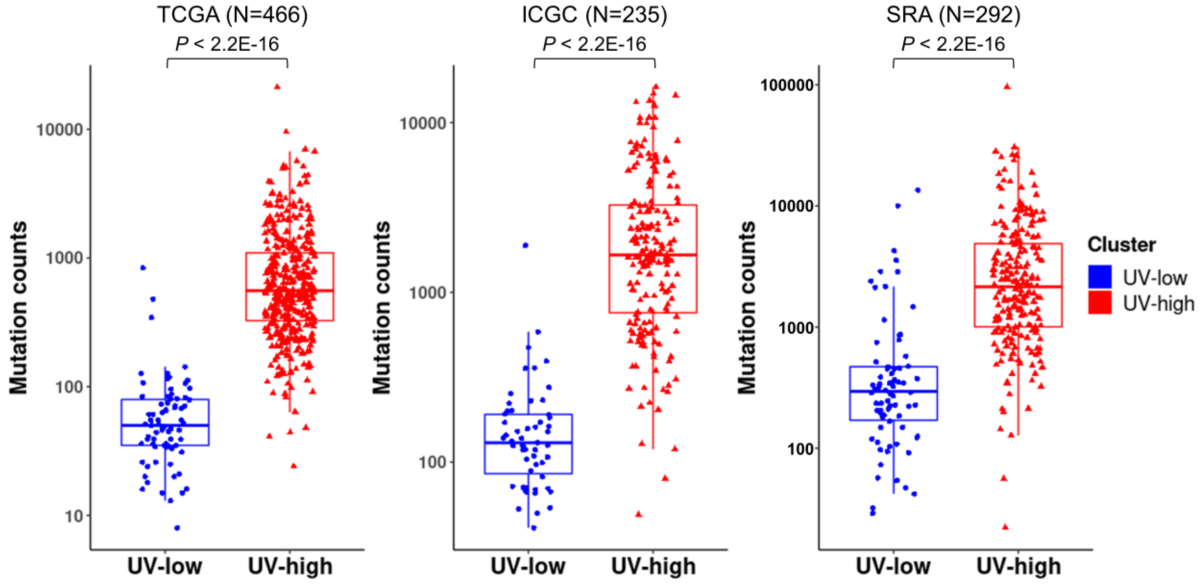

Supplementary Figure S7. Principal component analysis plot of mutational context of three datasets (TCGA, ICGC, and SRA). Principal component analysis of mutational context found that UV-low and UV-high cluster showed distinct distribution in all three datasets.

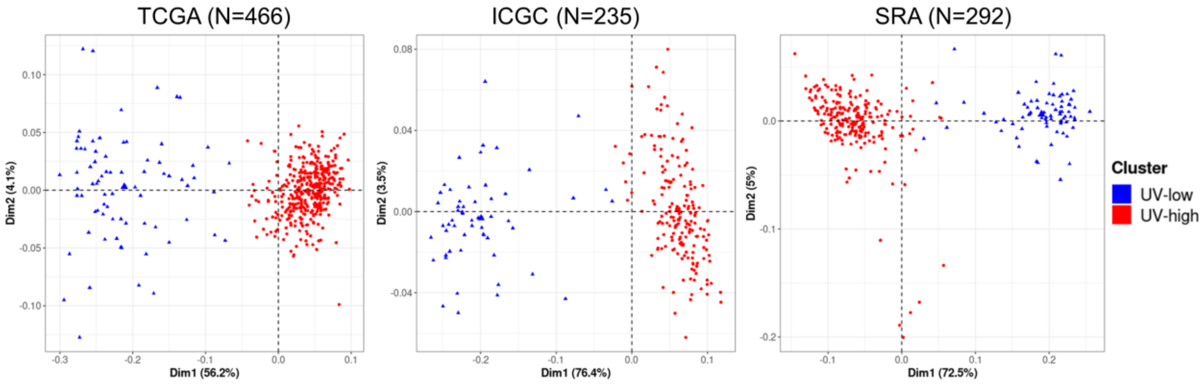

Supplementary Figure S8. Comparison of mutational context of all variants and variants within exonic region. In MELA-AU study (whole-genome sequencing dataset), high cosine similarity (median cosine similarity 0.977) were found between 96-class mutational context of all variants and the variants in exonic region (*in silico* down-sampled), although mutation counts in exonic region were reduced to 1.99% of one in whole-genome.

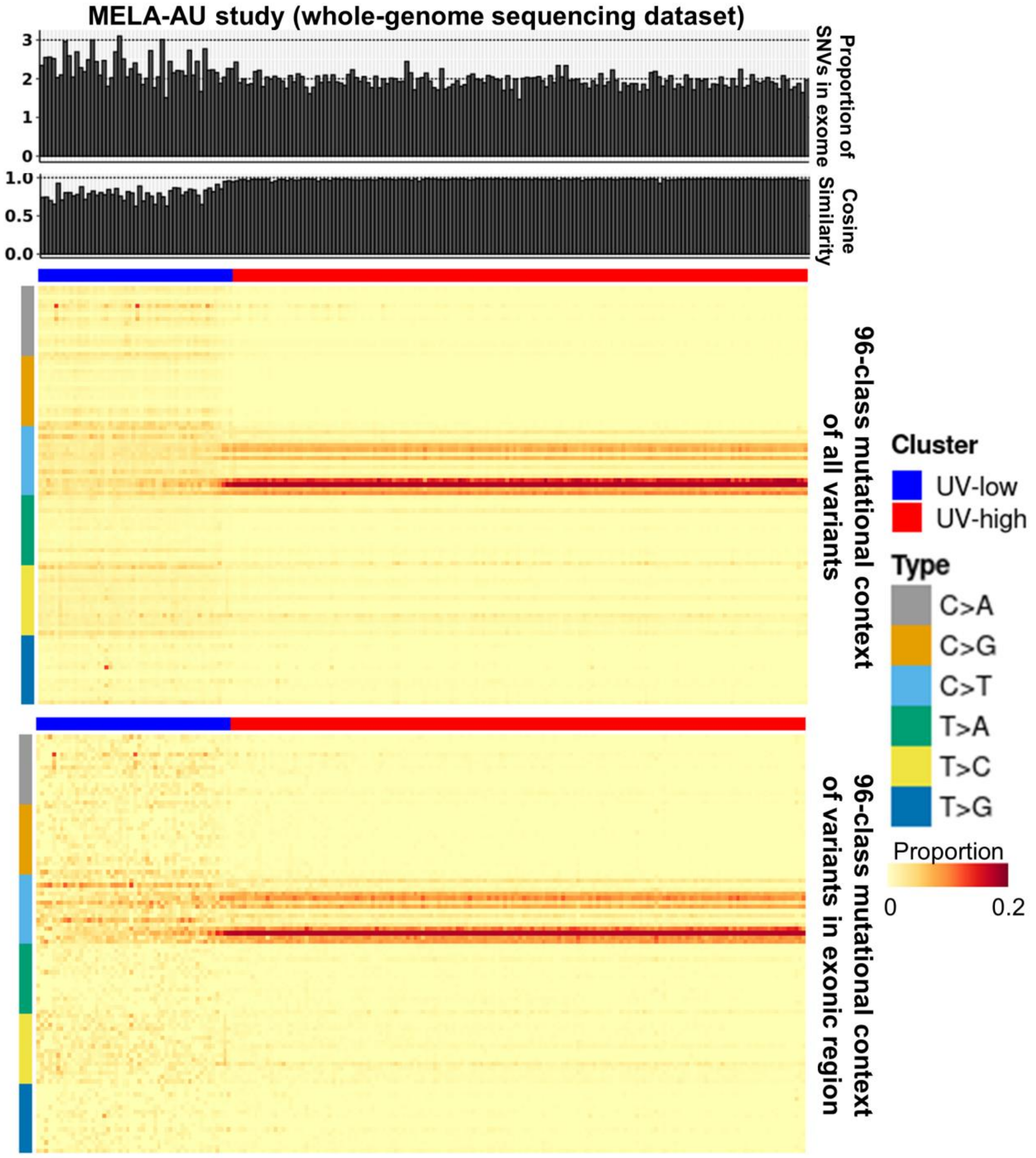

Supplementary Figure S9. Signature refitting analysis in ICGC and SRA dataset (A) Signature refitting analysis of the ICGC dataset (left panel) and SRA dataset (right panel) by known COSMIC signatures operating in melanoma. Samples (column) were arranged by the weight of SigB. (Same order as **Figure S5**).

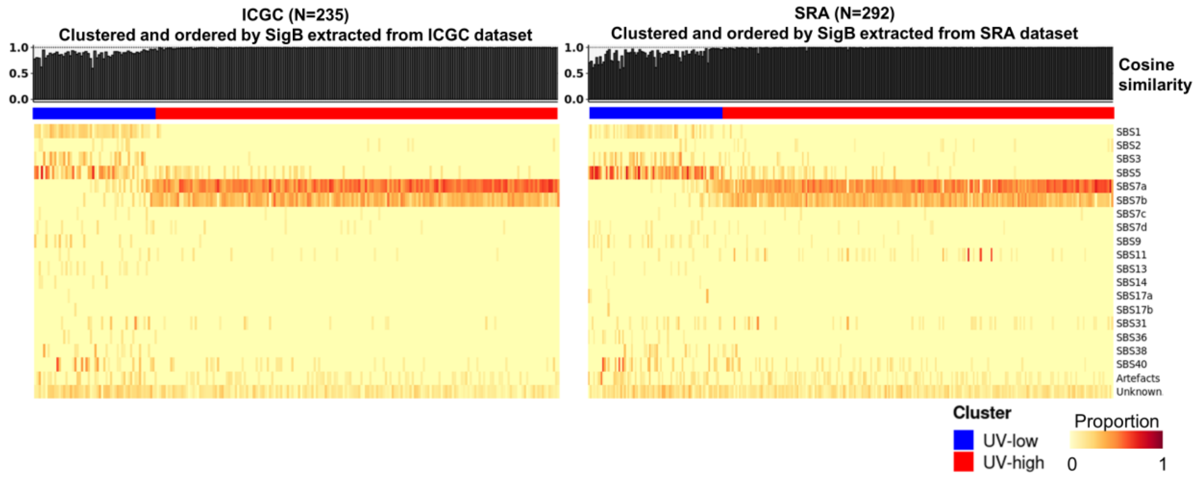

Supplementary Figure S10. Clinical significance of mutational signatures in TCGA cohort (A) Kaplan–Meier curves for UV-low (blue) and UV-high (red) in subgroups: stage I/II (N = 228), stage III/IV (N = 189), age > 40 (N = 391), *BRAF* hotspot (N = 199), and non-*BRAF* hotspot (N = 252). UV-low group showed significantly poorer prognosis than UV-high group in all subgroups (P < 0.05) except for *BRAF* hotspot (P = 0.059). (B) UV-low cluster showed higher proportion of advanced clinical stage (stage III/IV) (P = 0.024), ulceration (P = 0.001) and higher invasion depth (Clark level IV/V) (P = 0.002), compared to UV-high cluster. P-value was calculated using a two-sided Chi-square test.

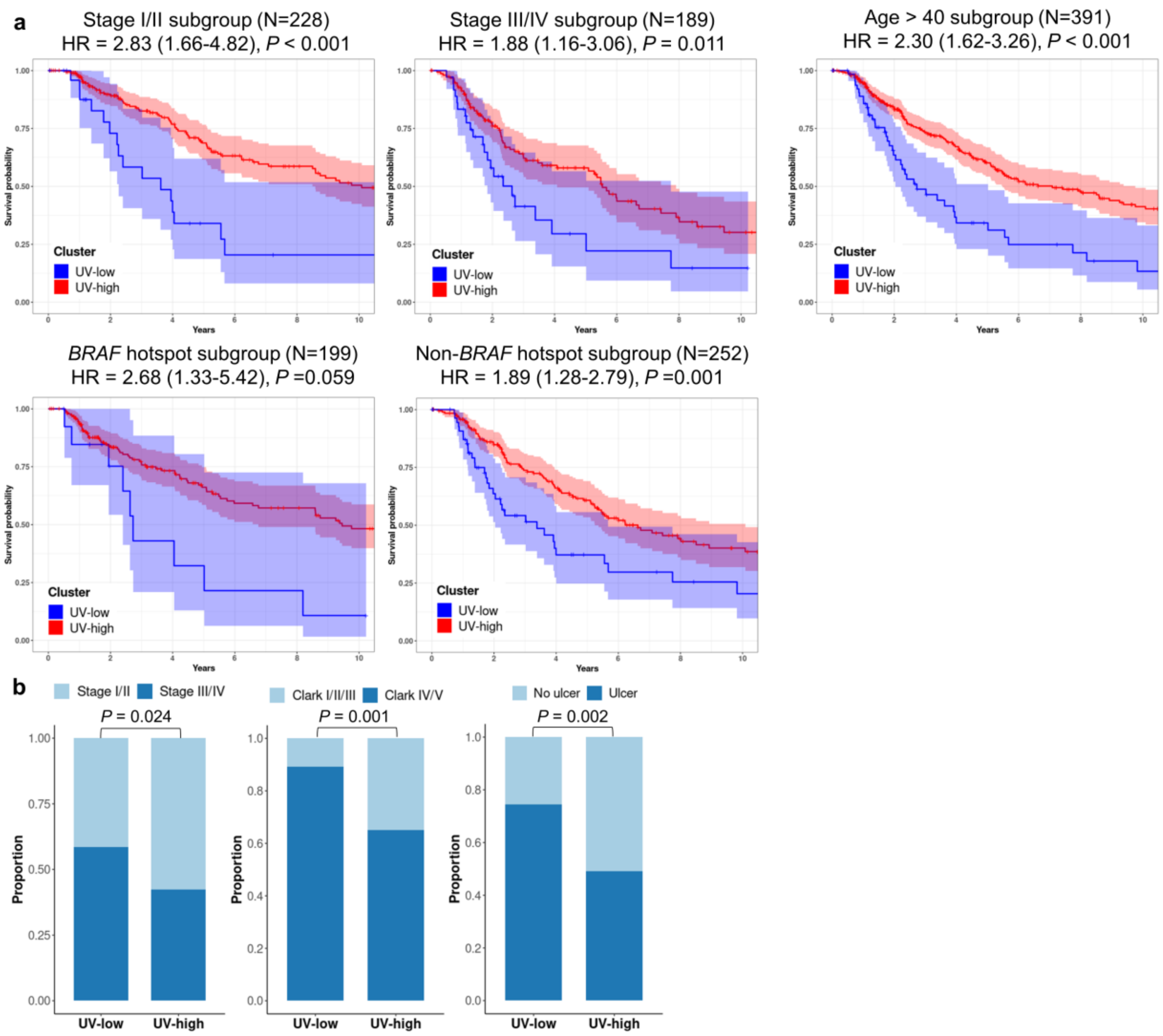

Supplementary Figure S11. *In silico* immunoprofiling analysis from RNA expression profiles using CIBERSORTx tool. Lower number of total immune cell (P = 0.007) and specific immune cells related to tumor immunity including macrophage M1 ($P < 0.001$), CD4+ memory activated T cells (P = 0.001), regulatory T cells (P = 0.008), and CD8+ T cells (P = 0.027) were found in UV-low cluster, compared to UV-high cluster. P-value was calculated using a two-sided Mann-Whitney U test.

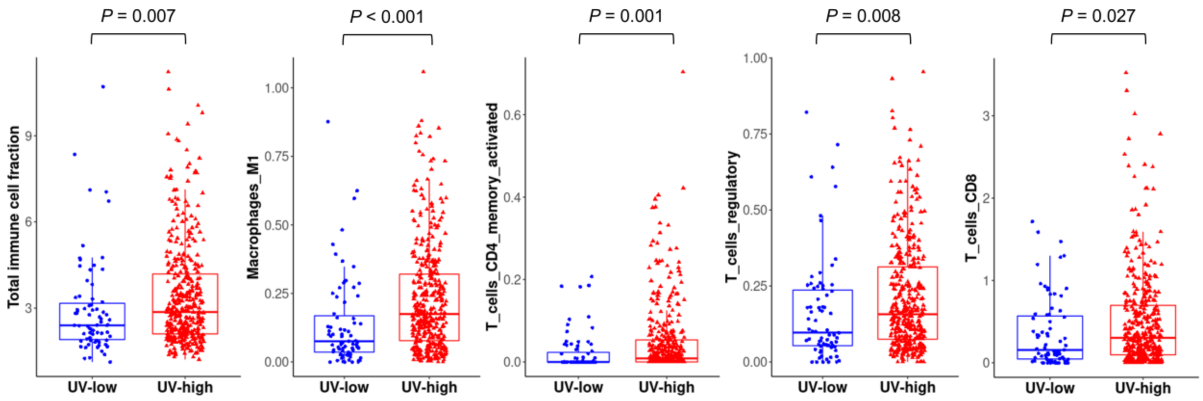

Supplementary Figure S12. Comparison of the signature-based clustering with known subtypes from TCGA study (UV, mutation, expression, miRNA, methylation, and protein). Bar plot shows the proportion of known subtypes from TCGA study (UV, mutation, RNA, miRNA, methylation, and protein) in UV-low and UV-high clusters. The number of patients with available information of subtype was shown below the name of subtypes. *P*-value was calculated using a two-sided chi-square test. Asterisk indicates significantly higher proportion of corresponding variables according to the *P*-values (*, $P < 0.05$; **, $P < 0.01$, ***, $P < 0.001$).

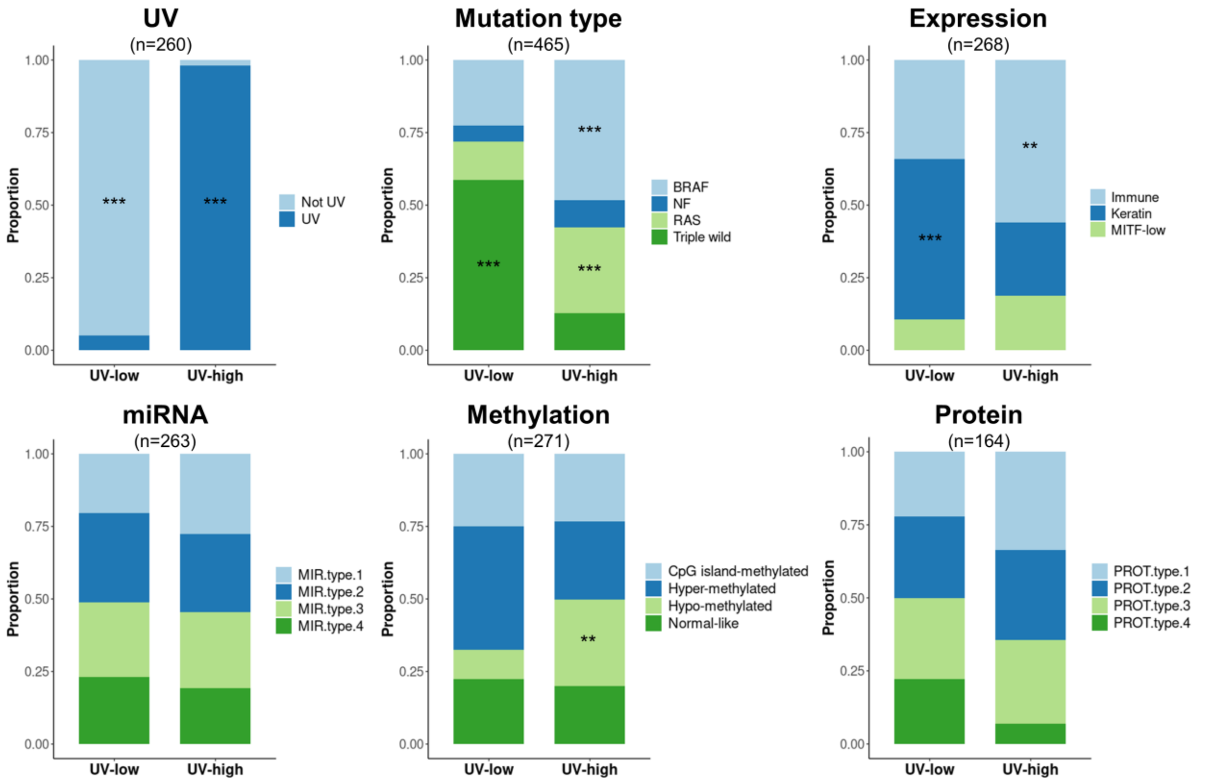

Supplementary Figure S13. Comparison of mutational context of WES and *in silico* panel simulation in TCGA cohort. Mutational context of (A) WES and (B) *in silico* panel simulation, proportion of mutation remained after simulation, and cosine similarity between mutational context of WES and simulation were shown. Samples (column) were arranged by the weight of SigB (Same order as **Fig. 1b** after excluding samples with less than 5 SNVs).

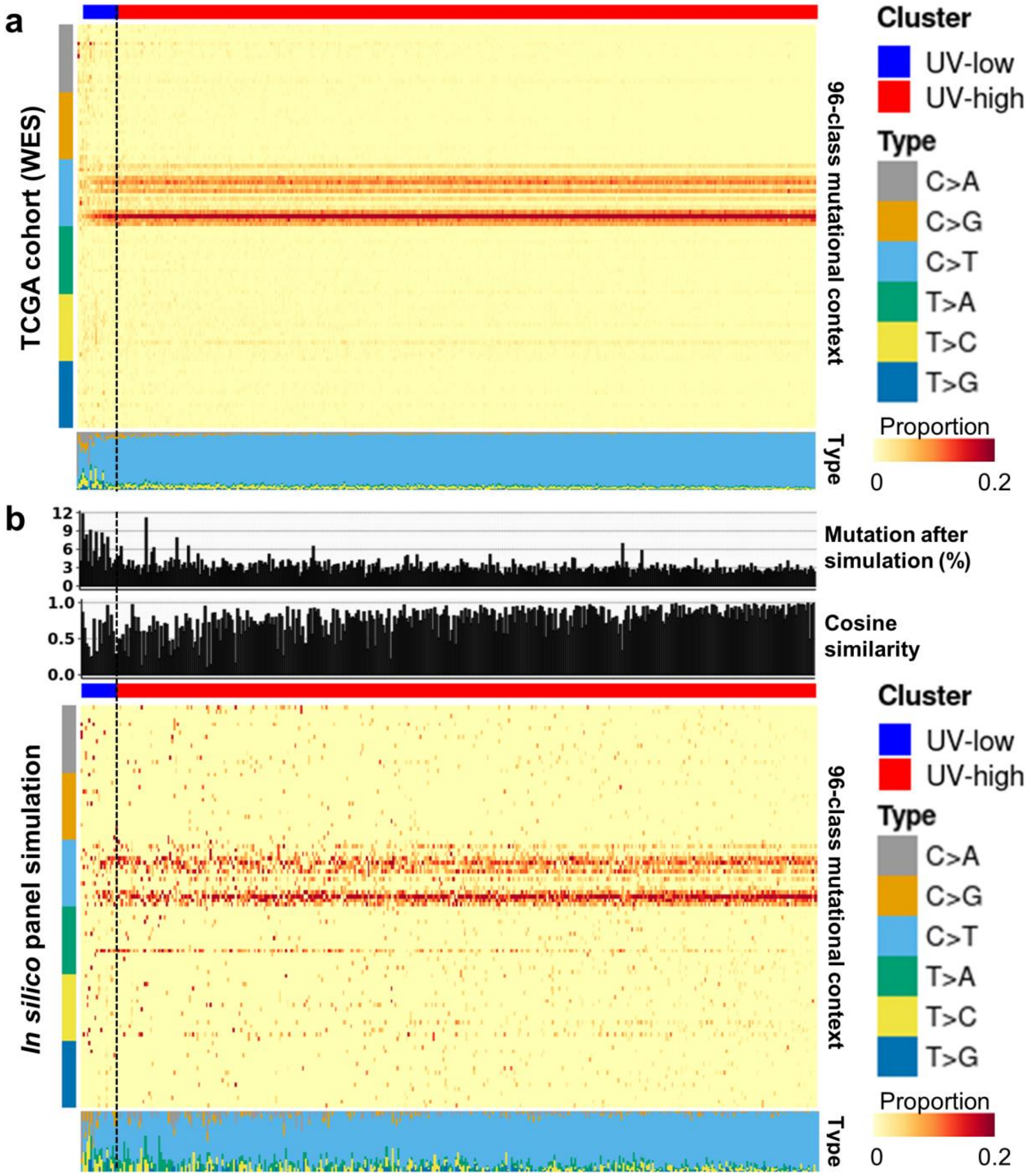

Supplementary Figure S14. Panel sequencing cohort (MSK cohort) classified by dominant signatures. Samples in panel sequencing cohort (MSK cohort) were classified by the dominant signatures: UV-low and UV-high clusters. Mutation count and mutational context were distinct between UV-low and UV-high clusters. Samples (column) were arranged by the weight of SigB (Same order as **Fig. 5c**). Samples with less than 5 SNVs were excluded.

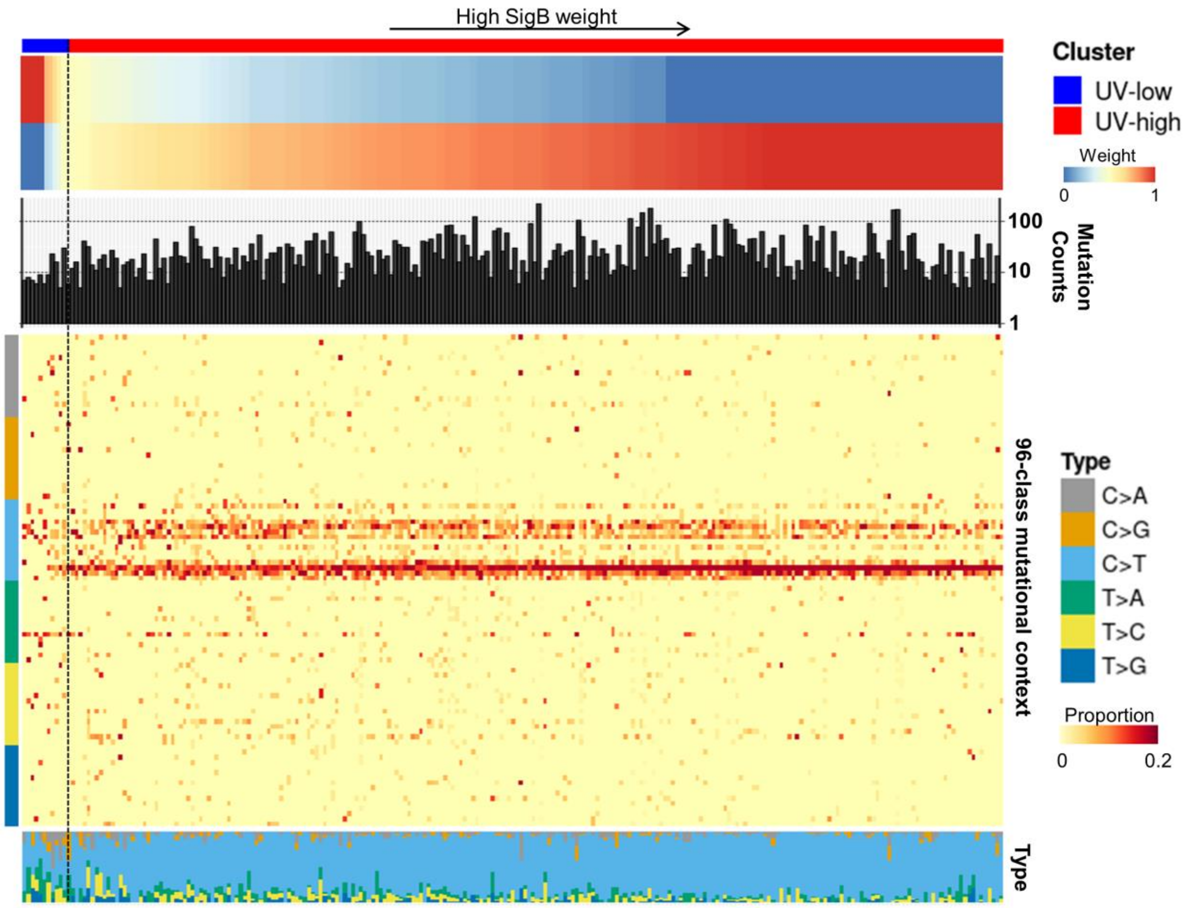

Supplement: Supplementary file 1 [file DataSheet1.pdf]
